# Effects of Hyaluronan on Breast Cancer Aggressiveness

**DOI:** 10.3390/cancers15153813

**Published:** 2023-07-27

**Authors:** Arianna Parnigoni, Paola Moretto, Manuela Viola, Evgenia Karousou, Alberto Passi, Davide Vigetti

**Affiliations:** Department of Medicine and Surgery, University of Insubria, 21100 Varese, Italy; arianna.parnigoni@uninsubria.it (A.P.); paola.moretto@uninsubria.it (P.M.); manuela.viola@uninsubria.it (M.V.); jenny.karousou@uninsubria.it (E.K.); alberto.passi@uninsubria.it (A.P.)

**Keywords:** hyaluronan, extracellular matrix, HAS2-AS1, HAS2, proteoglycans, tumor microenvironment

## Abstract

**Simple Summary:**

Breast cancer is the most common neoplasm in women. Although the primary tumor does not appear in a vital organ, lethality is due to the ability of tumor cells to invade and seed distant organs, causing metastases. Approaches to reduce breast cancer cell aggressiveness target hormone receptors that sustain cell growth and motility. However, other factors contribute to aberrant cell behaviors in cancer cells, and nowadays, the role of the environment surrounding cancer cells is evident. The extracellular matrix polysaccharide hyaluronan is a ubiquitous component of the tumor microenvironment that not only modulates cell growth and movement but also plays a critical role in modulating the inflammatory response. In this review, we discuss the role of hyaluronan in relation to the expression of critical hormone receptors.

**Abstract:**

The expression of the estrogen receptor (ER), progesterone receptor (PR), and human epidermal growth factor receptor 2 (HER2) in breast cancer cells is critical for determining tumor aggressiveness and targeting therapies. The presence of such receptors allows for the use of antagonists that effectively reduce breast cancer growth and dissemination. However, the absence of such receptors in triple-negative breast cancer (TNBC) reduces the possibility of targeted therapy, making these tumors very aggressive with a poor outcome. Cancers are not solely composed of tumor cells, but also include several types of infiltrating cells, such as fibroblasts, macrophages, and other immune cells that have critical functions in regulating cancer cell behaviors. In addition to these cells, the extracellular matrix (ECM) has become an important player in many aspects of breast cancer biology, including cell growth, motility, metabolism, and chemoresistance. Hyaluronan (HA) is a key ECM component that promotes cell proliferation and migration in several malignancies. Notably, HA accumulation in the tumor stroma is a negative prognostic factor in breast cancer. HA metabolism depends on the fine balance between HA synthesis by HA synthases and degradation yielded by hyaluronidases. All the different cell types present in the tumor can release HA in the ECM, and in this review, we will describe the role of HA and HA metabolism in different breast cancer subtypes.

## 1. Introduction

Breast cancer remains a significant global health concern, as almost 25% of cancer cases among women are breast cancer incidents [1]. Breast cancer is a heterogeneous disease consisting of different entities affecting the same anatomical organ and originating in the same anatomical structure (i.e., the terminal duct-lobular unit). Heterogeneity represents the primary challenge in treating breast cancer [2], and understanding the critical biomarkers, together with their role in carcinogenesis, drug resistance, and their use for diagnosis and therapy, is crucial for effective breast cancer treatment. The traditional histological classification of breast carcinomas, based on the diversity of the morphological features of the tumors, has the main drawback that approximately 80% of all breast cancers will eventually belong to either one of the two major histopathological classes, namely invasive ductal carcinomas or invasive lobular carcinoma. This implies that tumors with very different biological and clinical profiles are grouped, resulting in minimal prognostic and predictive capabilities and modest clinical utility [3]. To compensate for the limited prognostic and predictive power of the histopathological classification of breast carcinomas, Perou et al. introduced a new method of clustering breast tumors depending on their gene expression profiles and the presence or absence of estrogen receptors (ER), progesterone receptors (PR), and human epidermal growth factor receptor 2 (HER2). In such a way, breast carcinomas can be classified into five main molecular subtypes: luminal A (ER^+^/PR^+^/HER2^−^ with low or intermediate differentiation), luminal B (ER^+^/PR^−^/HER2^+^ with high differentiation grade), basal-like (ER^−^/PR^−^/HER2^−^) also called triple-negative breast cancer (TNBC), HER2-enriched (ER^−^/PR^−^/HER2^+^), and normal breast-like (overexpressing genes of adipose and other nonepithelial cells) [4,5]. In addition to these five, another class, called Claudin Low, was discovered, characterized by low gene expression of the tight junction proteins claudin 3, 4, 7, and E-cadherin [6].

Almost 70% of breast cancers are ER^+^ and hormone-dependent, making ER expression an optimal prognostic marker for responsiveness to treatment [7,8]. ER^+^ breast cancer is generally associated with a lower risk of recurrence, longer overall survival, and eventually a better prognosis than ER^−^ breast cancer. This difference is mainly attributed to the availability of hormonal therapies for ER^+^ breast cancer, including aromatase inhibitors, selective estrogen receptor modulators (SERMs), and selective estrogen receptor degraders (SERDs) [9]. In contrast, lacking ER expression, TNBC patients are more predisposed to adverse outcomes, recurrence, and metastasis than those affected by other breast cancer subtypes [10,11,12]. Their treatment mainly relies on cytotoxic chemotherapy, either before (neoadjuvant) or after surgery (adjuvant), which is inadequate, even though TNBC cases often benefit from chemotherapy to a greater degree than other breast cancer subtypes [13]. Interestingly, ERα silencing in MCF-7 cells enhanced cell proliferation, migration, and invasion, and thus, epithelial-to-mesenchymal transition (EMT), by changing the expression of critical matrix effectors [14]. The lack of ER, PR, and HER2 expression makes the development and use of TNBC’s targeted therapies highly challenging. Apart from anthracyclines (such as doxorubicin) and taxanes (such as paclitaxel), poly (ADP-ribose) polymerase (PARP) inhibitors (such as olaparib) and immunotherapy (such as atezolizumab) have been approved for use in combination with chemotherapy [15,16,17].

The major problem with breast cancer is its tendency to form metastases. Surgical removal of the primary site of the tumor is often insufficient to avoid distant metastases that typically develop in the liver, bones, lungs, and brain. EMT is the process enabling the development of more invasive tumors that, indeed, are characterized by a mesenchymal-like phenotype [18]. During EMT, epithelial tumor cells lose their cell-cell adhesion, dedifferentiate, gain a mesenchymal phenotype, and secrete an abnormal amount of extracellular matrix (ECM) in a process that highly resembles wound healing. Thus, cells acquire both invasive and metastatic capacities, increasing cell motility, invasiveness, and resistance to apoptosis [18,19,20]. These changes allow cells to detach from the primary tumor, invade the surrounding tissues, intravasate into the blood or lymphatic vessels, and ultimately form secondary tumors at distant sites, a process known as metastasis.

The tumor niche plays a pivotal role in breast cancer progression and metastasis [21]. The migratory behavior of cells and EMT itself are driven not only by the expression of a plethora of transcription factors (i.e., the snail/slug family, twist, EF1/ZEB1, SIP1/ZEB2, and E12/E47) [22], but a crucial role is also played by the ECM [23]. Depending on its composition, the chemical and mechanical properties of the ECM can vary, thereby influencing cell behavior. The ECM not only represents a scaffold on which cells attach and grow but also behaves as a sort of cage, trapping cells and limiting their movements. Interestingly, the degradation of ECM makes available growth factors that are normally bound to ECM components, such as proteoglycans (PG), and fragmented ECM molecules can have signaling activity that stimulates cancer cells [24]. Therefore, it is evident that ECM components play a crucial role in tumor biology.

Among the different components of ECM, this review focuses on the role of hyaluronan (HA) in relation to the aggressiveness of breast cancer cells.

## 2. The Role of HA in Tumor Stroma

HA, a prominent component of the ECM that is almost ubiquitously present in all vertebrates tissues, is an atypical glycosaminoglycan (GAG), as it is not covalently bound to any PG core protein, lacks any chemical modification, and is the only one produced outside the Golgi [25]. Indeed, the enzymes HA synthase 1, 2, and 3 (HAS1, 2, and 3) are located on the cellular membrane and directly extrude the nascent HA molecule outside the cells. HA polymers consist of repeating disaccharide units of D-glucuronic acid (GlcUA) and N-acetyl-D-glucosamine (GlcNAc), linked together by alternating β-1,4 and β-1,3 glycosidic bonds [26].

**Figure 1:**
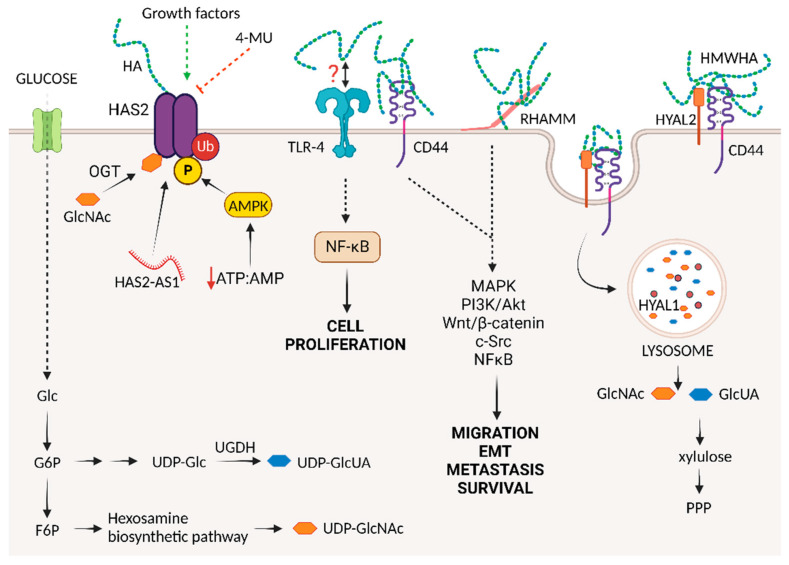
Schematic representation of hyaluronic acid (HA) synthesis, metabolism, degradation, and signaling via hyaladerin interactions. In cancer, HA synthesis is mainly sustained by HA synthase 2 (HAS2), whose activity of is controlled by several growth factors and post-translational modifications, including ubiquitination, O-GlcNAcylation, and phosphorylation, the latter being induced by a decrease in ATP:AMP ratio. Moreover, HAS2 antisense 1 (HAS2-AS1) long noncoding RNA (lncRNA) epigenetically regulates HAS2 transcription. Pharmacological agents, such as 4-methylumbelliferone (4-MU), prevent HA synthesis by downregulating HAS2 activity. Altered glucose metabolism enhances the accumulation of UDP sugars, thereby increasing HA synthesis. At the plasma membrane, HYAL2 chops high-molecular-mass HA (HMWHA) into small fragments that are soon internalized into the tumor cell by endocytosis through coordinated binding with CD44 and further degraded in the lysosomes by HYAL1. The resulting sugars, N-acetyl-D-glucosamine (GlcNAc), and D-glucuronic acid (GlcUA), can be recycled via cell energy metabolism. HA in the tumor microenvironment can interact with different membrane receptors, including CD44 and RHAMM, thus stimulating protumorigenic pathways supporting cell proliferation, migration, invasion, and metastasis. HA also stimulates cell proliferation via inducing a toll-like receptor 4 (TLR-4)-dependent signaling cascade, although HA interaction with TLR receptors has never been demonstrated. Abbreviations: Glc, glucose; G6P, glucose-6-phosphate; F6P, Fructose-6-phosphate; PPP, pentose phosphate pathway.

HA is ubiquitously present in all ECM tissues, showing many important physiological and pathological functions. The best-known role of HA is its structural function; HA acts as a chemical glue, allowing the formation of a stable ECM through the ability to interact with several proteoglycans (such as aggrecan and versican) [27]. Such HA complexes determine tissue mechanical properties, such as flexibility and stiffness. HA binds to several receptors, such as CD44, the HA-mediated motility receptor (HMMR, RHAMM, or CD168), and Lyve-1. It is known that the interaction of HA with these receptors can influence survival, proliferation, and motility through signaling cascades (Figure 1).

HA is synthesized as a high molecular mass polysaccharide with an average size of 4 × 10^6^ Da, and its turnover is assured by a complex family of degrading enzymes, including HYALs, PH-20, CEMIP, and regulatory proteins such as TMEM2 [28,29,30]. HA fragments are known to have distinct cellular functions compared to the full-size molecule; high-molecular-mass HA (HMWHA; >500 kDa) inhibits, whereas low-molecular-mass HA (LMWHA; <120 kDa) promotes angiogenesis, inflammation, and proliferation [31,32,33,34] (Figure 1). However, some exceptions have been observed; specific LMWHA is critical for proper gut development [35]. Notably, LMWHA can be generated by impaired homeostasis, HAS dysregulation, UV light, or oxidative stress [36]. HA accumulation is detected wherever rapid tissue remodeling and repair are present, that is, during embryogenesis, wound healing, inflammation, and even tumorigenesis. Among all the matrix components, HA is one of the most deregulated in human malignancies, and its amount in the tumor stroma affects overall survival and outcome [37,38,39]. High levels of HA are observed in several tumors compared to healthy tissues, such as renal [40], hepatocellular [41], head and neck [42] and lung squamous cell carcinomas [43], lymphoma [44], glioma [45], prostate [46], melanoma [47], breast [48], and ovarian [49] cancers. In most cancer cases, stromal accumulation of HA strongly correlates with an unfavorable prognosis and decreased survival probability [37,49,50]. As previously highlighted [51,52,53,54], obesity and type-2 diabetes are both risk factors for breast cancer. In particular, breast cancer in fatty breasts generally contains high levels of M2-like tumor-associated macrophages, both derived from the general state of inflammation of the tissue and their recruitment [55] and polarization towards the M2-phenotype due to cable-like HA [56].

HA involvement in supporting tumor metastasis has been widely reported; remarkably, other than driving tumor cells into the endothelium, LMWHA also supports endothelial budding and capillary formation in 3D matrices, thus increasing breast tumor angiogenesis and lymphangiogenesis [36,57].

The aberrant accumulation of HA in malignant tissues is mainly due to the deregulated activity of HAS, hyaluronidases, reactive oxygen species (ROS), and hyaladerins (mainly CD44) [58]. Moreover, it must be considered that HA is synthesized not only by cancer cells; stromal cells are also stimulated to produce a high amount of HA under the stimulation of factors released by cancer cells themselves, as well as infiltrating immune cells, cancer-associated fibroblasts, and others [21,49,59,60,61,62]. The first studies demonstrating that stromal cells produce HA upon interaction with tumor cells date back to the 1980s [63,64]. Notably, cancer-associated stromal cells can stimulate epithelial cells to transform into malignant cells owing to the release of growth factors, chemokines, and cytokines, including fibroblast growth factor (FGF), platelet-derived growth factor (PDGF-BB), transforming growth factor β (TGFβ), interleukin-6 (IL-6), IL-8, and chemokine (C-X-C motif) ligand 7 (CXCL7) [21]. Currently, the involvement of HA in tuning the Hanahan and Weinberg hallmarks of cancer is well accepted and has become a very promising target in cancer research [65,66].

## 3. HA Synthesis and Metabolism

HASs are the main regulators of HA synthesis, possessing all the enzymatic activities to produce the HA polysaccharide chain and extrude it into the ECM. Indeed, they have a single catalytic domain, which transfers two different donor sugars and forms substrate-specific glycosidic linkages, and a transmembrane channel derived from the spatial organization of their membrane-embedded segment, through which the nascent HA chain is secreted into the extracellular space [67]. Recently, the structure of HAS was analyzed using cryoelectron microscopy, shedding light on how HAS selects its substrates, hydrolyzes the first substrate to prime the synthesis reaction, opens an HA-conducting transmembrane channel, ensures alternating substrate polymerization, and coordinates HA inside the channel, confirming the “pendulum” model proposed by Weigel et al. [68,69].

Humans express three different HAS isoforms, derived from three related and evolutionarily highly conserved genes, yet located on three different chromosomes, differing in tissue expression and catalytic activity [70].

An important feature of all HASs is their capability to synthesize HA starting from two UDP-sugar precursors, exploiting the high energy content of the two substrates and thus avoiding the need for ATP [71]. Interestingly, although ATP is not directly involved in HA production, the synthesis of the two UDP-sugar precursors (i.e., UDP-GlcUA and UDP-GlcNAc) is affected not only by the energetic status of the cells but also by other metabolic pathways. The double oxidation of carbon 6 of UDP-glucose to produce UDP-GlcUA leads to the production of two NADH molecules that influence the NAD:NADH ratio, which is critical for the activity of a variety of enzymes, including dehydrogenases and sirtuins [25,26,72]. UDP-GlcUA is an important substrate for glucuronyltransferase detoxifying enzymes [73]; therefore, an increase in UDP-GlcUA contributes to enhanced chemoresistance in aggressive cancers. Together with UDP-GlcUA, UDP-glucose dehydrogenase (UGDH), which is responsible for the conversion of UDP-glucose into UDP-GlcUA, has an important role in drug detoxification. Interestingly, its knockdown in MDA-MB-231 cells contributed to epirubicin chemoresistance, which is associated with increased deposition and catabolism of HA [74] (Figure 1).

UDP-GlcNAc is synthesized by the hexosamine biosynthetic pathway, which integrates the metabolism of sugars, amino acids, fatty acids, and nucleotides. Therefore, in addition to the presence of HASs, the availability of UDP-GlcUA and UDP-GlcNAc is critical for proper HA biosynthesis. Cancer cells show a deep alteration in metabolism, which can greatly modify HA biosynthesis [66]. Indeed, aggressive breast cancer tumors, characterized by deeply disturbed glucose metabolism, have a higher amount of UDP-sugars, which eventually influence the cancer niche by inducing HA production and deposition without affecting the expression levels of any of the HAS [75].

The first stages of tumorigenesis are characterized by a limited oxygen supply, and the hallmark of breast cancer cells is the Warburg effect, which is dominated by an increased flux of glucose through anaerobic glycolysis to generate ATP and lactate [76]. Although experimental data in this early phase of cancer development are scarce, it can be hypothesized that HA synthesis in neoplastic cells is low. Firstly, glucose should be mainly channeled into catabolic processes rather than shunted to anabolism; second, the NADH produced during the oxidation of UDG-glucose to UDP-GlcUA could be difficult to convert to NAD in the absence of oxygen. Interestingly, the elevated HA in cancer stroma could be produced by fibroblasts, as lactate stimulates HA synthesis in this kind of cell [77]. HA is important for breast cancer cells because it ensures efficient lactate efflux [78]. Furthermore, several HA-metabolizing genes, such as *CD44* and *HYAL1*, possess lactate-responsive elements [79], which could favor rapid HA turnover leading to the formation of important building block intermediates (such as ribose and NADPH; see later in Section 4—HA catabolism) needed for rapidly growing cells. In contrast, in the late stages of tumorigenesis, when angiogenesis happens, oxygen availability is no longer limited, and aggressive breast cancer cells can produce an elevated amount of HA, thus favoring several aspects of tumor malignancy, including motility and stemness [80].

### 3.1. The Role of HASes in Breast Cancer

The presence of three HAS isoenzymes in humans remains unclear, as their exact role in pathophysiology is only partially known. What is known is that they have distinct spatial and temporal expressional and transcriptional patterns and regulators [81,82,83]. Moreover, each HAS can synthesize HA chains of different sizes; HAS1 and -2 catalyze the formation of HMWHA, whereas HAS 3 favors the production of LMWHA [34]. Although all three HASs are found in various tumor cell types and both HAS2 and HAS 3 expression is correlated with malignant transformation, HAS2 is the most efficient HA-synthesizing enzyme [30,84]—its expression is fundamental during embryogenesis, as HAS2-deficient mice result in embryonic lethality [85], while HAS1 and HAS3 deletions have only minor effects on the phenotype [81]. HAS1 expression is generally low in normal breast tissue but is upregulated in breast cancer. Its overexpression enhances the metastatic potential of breast cancer cells [86] and correlates with shorter overall survival, a higher relapse rate, ER negativity, and HER2 positivity [87] (Table 1). Interestingly, its localization seems to be mostly cytoplasmic, where it cooperates in promoting breast cancer cell growth, intratumoral heterogeneity, and a cancer stem cell-like phenotype [88,89].

Several studies have highlighted the predominant transcriptional activity of the *HAS2* gene in ER^−^ aggressive breast cancer cell lines compared to the limited expression of HAS3 in nonaggressive ER^+^ cell lines [91,92,93] (Table 1). It has to be highlighted that stromal cells play a pivotal role in producing abnormal amounts of HA, and in these cells, HAS1, and not HAS2, is the strongest isoform affecting tumor relapse and patient prognosis [87]. In breast cancer, both high HAS2 and HA levels have been shown to induce the recruitment of tumor-associated macrophages (TAM) and tumor neovascularization; stromal HA, via macrophage recruitment, remodels the local microenvironment to promote the formation of tumor vasculatures [94,95]. In TNBC (i.e., MDA-MB-231 and Hs578T), HAS2 mRNA levels are significantly higher than those in less aggressive ER^+^ cell lines (i.e., MCF-7) [96] (Table 1). Notably, HAS2 overexpression has also been correlated with TGFβ-mediate EMT [97].

Furthermore, HAS2 also plays a pivotal role in regulating cell motility and invasion, as its expression levels are higher in bone metastases than in parental MDA-MB-231 cells [93]. HAS2 expression was also found to be elevated in the cancer stem cell population of breast cancer bone metastasis, and its suppression decreased both the incidence and growth of metastatic lesions [98]. In the ER^+^ breast cancer cell line, overexpression of HAS2 increased the invasive and migratory abilities of cells, together with the downregulation of epithelial markers (e.g., E-cadherin, β-catenin, and ZO-1), upregulation of mesenchymal markers (e.g., N-cadherin and vimentin), and promotion of invadopodia formation [99]. Finally, breast tumor biopsies showed enhanced angiogenesis and the recruitment of inflammatory cells when HAS2 expression was elevated. In support of these findings, HAS2 suppression by interfering RNA or 4-methylumbelliferone (4-MU) administration reduces tumorigenesis [91,100,101].

### 3.2. HAS Inhibition by 4-MU

Furthermore, 4-MU is a specific inhibitor of HAS activity, as it reduces the availability of UDP-GlcUA [102] and, indirectly, lowers HAS expression [103,104]. Its effectiveness in reducing tumor growth and aggressiveness has been reported in different cancer types [105,106,107,108]. Interestingly, 4-MU has been shown to reduce HA synthesis and HAS2 and CD44 expression, but increases HYAL1 and HYAL2 in breast cancer cell lines, and to a greater extent in ER^−^ cells. Moreover, only ER^−^ cell lines showed reduced migration, adhesion, and invasion [101]. However, 4-MU treatment is not specific for any particular HAS, and can partially affect the synthesis of other GAG chains [102]. It is reported that 4-MU causes potential detrimental effects, as described for glycocalyx alteration in atherosclerotic animal models [109] and increased tumorigenicity in hepatocellular carcinoma mouse models [110]. Recently, the potential limited systemic efficacy of 4-methylumbelliferyl glucuronide (4-MUG) was tested in mice. Additionally, 4-MUG reduced HA synthesis independently of its conversion into 4-MU without depleting the HA precursor UDP-GlcUA. However, 4-MUG is not specific to any HAS isoform [111].

### 3.3. HAS2 Regulations

HAS2 is the only enzyme that exhibits fine-tuned activity via post-translational modifications (Figure 1). Adenosine monophosphate-activated protein kinase (AMPK), a cellular energy sensor, induces the phosphorylation of HAS2 threonine 110, blocking its enzymatic activity and eventually connecting HAS2 activity with the energetic status of the cell [112]. AMPK generally activates catabolic processes to restore ATP levels and inhibit ATP-consuming pathways (i.e., anabolism). Since anabolic pathways are preferred to catabolic pathways in tumors, it is not surprising that AMPK is often found to be blocked in malignancies. In breast cancer, AMPK downregulation has been correlated with the loss of ER expression and a poor prognosis [113,114,115,116,117]. Liver kinase B1 (LKB1), an upstream activator of AMPK, is mutated in numerous malignancies, thus blocking the AMPK pathway and favoring HA synthesis [118]. Interestingly, salicylate has been demonstrated to induce AMPK, eventually downregulating HAS2 expression, HA production, and metastatic breast cancer cell proliferation by inducing cell growth arrest [119].

Other kinases, such as extracellular signal-regulated kinases (ERKs) and protein kinase C (PKC), seem to play a role in regulating HAS2 activity [120]. Finally, HAS2 can form dimers or oligomers upon monoubiquitination of the K190 residue, which plays a key role in its activity and dimerization [121].

More than being solely a precursor for HA synthesis, GlcNAc is also the substrate for O-GlcNAc transferase (OGT), which has a central role in the regulation of metabolism in response to nutrient availability. OGT transfers GlcNAc moieties to serine 221 of the HAS2 protein, increasing its stability in the plasma membrane (up to 5 h) and, thus, HA synthesis [122]. Interestingly, O-GlcNAcylation of the NF-κB subunit p65 plays a critical role in the induction of HAS2 transcription, involving HA synthase 2 antisense 1 (HAS2-AS1) long noncoding RNA (lncRNA) as a mediator of chromatin remodeling [122].

### 3.4. Epigenetic Regulation of HAS2

HAS2-AS1 was first described by Chao and Spicer in 2005 as the natural antisense of the HAS2 sequence, as it is located on chromosome 8q24.13 and is transcribed from the opposite strand of the HAS2 gene locus. In 2011, Michael and colleagues proved for the first time that under the stimulation of interleukin-1 β (IL-1β) and TGFβ, HAS2 mRNA physically interacts with HAS2-AS1 in proximal tubular epithelial cells, finally stabilizing and promoting HAS2 expression [123]. LncRNAs are known to have tissue-specific effects, and HAS2-AS1 is no exception [124]. Its overexpression in osteosarcoma cells reduces HAS2 transcript [125], while in oral squamous cell carcinoma, it induces HAS2 transcription and hypoxia-induced invasiveness [126]. In mouse mammary gland epithelial cells, HAS2-AS1 is known to induce cell transformation via TGFβ [127]. Although it is well accepted that HAS2-AS1 works as a cis epigenetic regulator of HAS2 in aortic smooth muscle cells (AoSMCs) [72,128], this mechanism of action seems to be invalid in breast cancer. Indeed, HAS2-AS1 expression was higher in TNBC cell lines (MDA-MB-231 and Hs 578T) than in ER^+^ cells (MCF-7 and T-47D), and its overexpression in MDA-MB-231 and Hs 578T cells reduced cell aggressiveness by inducing mesenchymal-to-epithelial transition and reducing invasiveness, motility, and cell viability (Table 1). Notably, in these cells, HAS2-AS1 was involved neither in the regulation of HAS2 nor in HA deposition. Surprisingly, its expression was higher in TNBC cell lines than in ER^+^ cells, and its expression directly correlates with ER^−^ breast cancer patients’ survival [90]. In addition to being solely an epigenetic regulator of the HAS2 gene, HAS2-AS1 can also be found in the cytoplasm [127]. Different studies have reported the intriguing ability of HAS2-AS1 to interact with other RNAs, acting as competing endogenous RNAs (ceRNAs) for miRNAs. An increasing number of studies have reported that HAS2-AS1/miRNA interactions are mainly functional in cancer [129,130].

HA synthesis can also be increased by golgins and, in particular, by the protein c10orf118, which is abundant in the conditioned media of breast cancer cell lines. c10orf118 induces HAS2 expression in dermal fibroblasts, thereby stimulating HA synthesis [131,132].

Some studies have reported the involvement of HAS2 in breast cancer plasticity and [133] chemotherapy drug resistance [134,135]. Notably, HAS2 knockdown in ER^+^ breast cancer cell lines induces upregulation of Ezrin, downregulation of ER and, thus, antiestrogen resistance [136].

## 4. HA Catabolism

In mammalian tissues, the length of the HA chain varies significantly [137]. The polydisperse sizing of HA mainly depends on the catalytic abilities of HYALs, which are hydrolases that cleave the β-(1,4) linkage between GlcNAc and GlcUA. There are six known hyaluronidases in humans: HYALs 1-4, HYALP, and PH-20 [138]. HYALs’ degrading activity is not only related to HA; HYAL4, for example, can cleave chondroitin sulfate, with no evidence of HA catabolic activity [139]. Among all HYALs, HYAL1, and HYAL2 are the predominant isoforms that cleave HA in cooperation with CD44. At the plasma membrane, HYAL2 chops HMWHA into small fragments that are soon internalized into the cells via endocytosis and further degraded in the lysosomes by HYAL1. Inside the cells, HA is completely degraded by the coordinated action of HYALs, β-glucuronidase, and hexosaminidase, leading to free GlcUA and GlcNAc [29]. Eventually, GlcUA is converted to xylulose-5-phosphate, which sustains the hexose monophosphate pathway for the synthesis of NADPH and ribose.

Recently, other HA-degrading enzymes have been reported to exhibit hyaluronidase activity, including the HA-binding protein involved in HA depolymerization (HYBID, also known as CEMIP) and TMEM2 [140,141]. In contrast to HYALs, which function at an acidic pH, TMEM2 functions at a physiological pH and, being regulated by SOX4, is known to be associated with metastatic migration and invasion of breast cancer cells [142]. CEMIP degrades HA after clathrin-dependent internalization into endosomes and is known to promote the progression and metastasis of numerous malignancies, including breast cancer [141,143,144,145].

The catabolism of HA results in the generation of bioactive fragments with contrasting size-dependent functions [146,147]. For instance, HMWHA is known to have antiangiogenic, antiproliferative, and immunosuppressive properties. On the other hand, LMWHA induces inflammation and angiogenesis, has immunostimulatory functions, and induces tissue reparative processes, as described in wound healing [137,148]. As proof, naked mole-rats, which possess HA chains longer than 12 million Da, have an unusually long life, approximately ten times longer than other rodents, and an incredible resistance to tumor development and spreading when cancer cells are injected into the dermis [149]. Recently, it was demonstrated that these animals also express very low levels of HYAL1 and HYAL3 [150].

### The Role of HA Oligosaccharides in Breast Cancer

Metastatic breast cancer is associated with an increased amount of serum LMWHA, and BT-549 and Hs578T TNBC cell lines have been found to produce a high amount of LMWHA, making it a good prognostic marker [151]. Contrary to what happens in the naked mole rat, tumors typically express high levels of HYALs. These enzymes also contribute to sustaining cancer metabolism and metabolic reprogramming, providing support to the pentose pathway to obtain reducing equivalents and ribose for anabolism and increasing the glycolytic rate to produce ATP [152]. Indeed, matrix remodeling through HYAL-mediated HA digestion increases glycolysis in several tumors, including breast cancer [153]. The combined overexpression of HAS and either HYAL1 [154] or HYAL2 [155] is characteristic of the invasive front of human breast cancer. TNBC cell lines have been shown to express high levels of HYAL2, HAS2, and CD44 [91] (Table 1).

In addition to being enzymatically degraded by HYALs, HA is also susceptible to ROS degradation [156,157]. Significant ROS production is typical in inflamed tissues, and cancer is a major inflammatory disease. Oxidative stress is simultaneously beneficial and detrimental for tumors; if it is essential in initiating and promoting malignant growth, an excess of oxidative stress could lead to the apoptosis of cancer cells. In this case, the abundant HA in the ECM acts as a protective shield against excessive reactive nitrogen species (RNS) and ROS. However, HA cleavage releases an excessive amount of bioactive fragments, promoting inflammatory pathologies, including cancer [156,158]. If HMWHA serves as a scavenger for ROS/RNS, HA fragments, especially those produced by ROS, act as danger signals, inducing the synthesis of cytokines, eventually exacerbating inflammation, and inducing classic tumor-associated pathways such as angiogenesis, as observed in breast cancer [159]. The presence of LMWHA in the serum of breast cancer patients is useful for discriminating between metastatic and nonmetastatic breast cancers [151].

## 5. Hyaladerins in Breast Cancer

The multiple and even contradictory effects of HA polymers of different sizes can be attributed to the presence of several HA receptors on the cell surface, which can trigger specific and differential signaling cascades. Among these, the most well-known are CD44, RHAMM, lymphatic vessel endothelial receptor 1 (LYVE-1), and the HA receptor for endocytosis (HARE) (Figure 1). It is also known that HA modulates toll-like receptors 2 and 4 (TLR2 and 4); however, HA binding to TLRs remains unclear [160]. Through the engagement of these binding partners, HA perturbs tissue homeostasis by driving cell motility, proliferation, apoptosis, and tissue remodeling.

The most prominent HA receptor is CD44, which is abundant in both inflammatory and cancer cells [160]. CD44 is a single-span transmembrane glycoprotein involved not only in mediating both cell-cell and cell-matrix interactions but is also a key player in transmitting HA signaling in tumor progression [161]. The peculiarity of the HA/CD44 interaction is that the receptor can acquire either a high- or low-affinity HA binding state. In humans, CD44 is encoded by a single gene that contains 20 exons, generating many variants (CD44v) and possessing different levels of glycosylation. The standard isoform of CD44 (CD44s) is ubiquitously expressed in all vertebrates, whereas CD44v is mainly expressed during inflammation and cancer [162]. Even though v6-v10 isoforms are most likely related to procancerous functions [163,164,165], CD44s have also been reported to induce EMT [166]. Notably, the switching between CD44s and CD44v plays a key role in regulating the EMT and plasticity of cancer cells [167]. Different breast cancer subtypes express peculiar CD44v—this diversity in CD44v expression has been ascribed to specific clinical markers (i.e., HER2, ER, PR), suggesting the involvement of CD44v in specific oncogenic signaling pathways [168]. Moreover, the CD44^+^/CD24^−^ breast cancer cell subpopulation, typically identified as cancer stem cells, is associated with invasive properties and a poor prognosis [169]. In fact, the HA/CD44 interaction promotes cytoskeletal remodeling, thus favoring cell growth, survival, invasion, and metastasis.

The HA/CD44 interaction involves the BX_7_B HA-binding motif in the N-terminal region of the CD44 receptor. HA interaction with CD44 triggers receptor clustering and interaction with other transmembrane proteins, including receptor tyrosine kinases, serine/threonine kinase receptors, tumor necrosis factor receptor (TNFR)-like receptors, G-protein-coupled receptor CXCR4, Wnt receptor LRP5/6, CD147, c-Met, VEGFR2, PDGF, IGF1R, ErbB family, TGFβ, and ATP-binding cassette transporters [57,167,170,171]. In such a way, the CD44 cytoplasmic domain can be phosphorylated to transduce signals when the ligand binds to the extracellular domain. The signaling cascade triggered by the HA/CD44 interaction mainly stimulates the phosphoinositide 3-kinase/phosphoinositide-dependent kinase-1/protein kinase B (PI3K/PDK1/Akt) pathway, the Ras phosphorylation cascade involving RAF1, mitogen-activated protein kinase (MEK), and ERK1/2, as well as Wnt/b-catenin, NF-κB, and focal adhesion kinases (FAK). Consequently, the HA/CD44 interaction stimulates several oncogenic pathways and microRNA (miRNA) functions related mainly to cell adhesiveness, migration, and infiltration [172].

The CD44 intracellular domain (CD44ICD) can undergo cleavage and then translocate into the nucleus, acting at least in part as a transcriptional regulator [173,174]. In breast cancer, CD44ICD induces the transcription of stemness factors such as Nanog, Sox2, and Oct-4, thereby contributing to breast cancer aggressiveness and tumorigenesis [175].

HAS2 and CD44 are known to be highly expressed in ER^−^ breast cancer, promoting tumor aggressiveness. An interesting study highlighted the strict correlation between HAS2 expression, HA/CD44 signaling, and ER-dependent tumor aggressiveness. Indeed, HAS2 overexpression reduces the estrogen dependence of MCF7 cells by inhibiting the transcription of ER-driven genes [176]. This mechanism could at least partially explain the aggressiveness of ER^−^ cells, where HAS2 and CD44 expression are higher than in ER^+^ cells. Moreover, the increase in HA/CD44 interaction promotes EMT by suppressing the anti-EMT effects of estradiol [177].

The second most important HA receptor is RHAMM (also known as CD168), a coiled-coil protein containing the BX_7_B motif that can be found on cell membranes, in the cytoplasm, nucleus, or extracellular space. High RHAMM expression has been reported in several tumors, including breast, colon, brain, prostate, and endometrial [178,179,180,181]. When binding HA, RHAMM can interact with other receptors, such as the PDGF receptor (PDGFR), TGFβ receptor I (TGFβRI), and CD44 [162]. Consequently, several pathways are induced, including Ras, FAK, ERK1/2, PKC, tyrosine kinase pp60 (c-Src), NF-κB, and PI3K, eventually leading to cell migration, wound healing, tumorigenesis, and EMT [182,183].

A large body of evidence supports the role of TLR2 and -4 in HA signaling. TLRs are membrane receptors typical of the immune system and are mainly related to inflammation during viral infection and tumorigenesis [184,185,186]. Recently, it has been demonstrated that HA interaction with both CD44 and TLR4 sustains colon tumorigenesis, by promoting tumor growth in mice [187]. Similarly, the HA/TLR4 interaction in glioblastoma provokes cell differentiation by activating NF-κB [188].

Another interesting HA-binding protein is TNF-stimulated gene 6 (TSG-6), which yields the formation of cross-links between HA and the heavy chains of the serine protease inhibitor inter-alpha-inhibitor (IαI), thus stabilizing the structural integrity of the ECM. In general, TSG-6 is not constitutively expressed in normal adult tissues, but its expression is upregulated during inflammation, inflammation-like processes (such as ovulation), and cancer progression [189,190]. TSG-6 can bind HA with high affinity, mediating the generation of HA cable-like structures and enhancing HA-CD44 binding, which facilitates leukocyte migration to the site of inflammation. In breast tumors, cancer-associated fibroblasts (CAFs) are responsible for ECM deposition in the tumor niche. Notably, in normal mammary tissues, normal fibroblasts deposit much more HA and TSG-6 than CAFs in breast tumors, where the levels of TSG-6 and, consequently, HA-TSG-6 cross-linked structures are significantly decreased and associated with higher tumor malignancy [191]. Moreover, the interaction between HMWHA and TSG-6 affects the angiogenic behavior of monocytes/macrophages [192].

## 6. Future Directions

The evidence that HA is a central player in breast cancer, able to fine-tune many aspects of cell aggressivity, is clear and solid and has been confirmed in several experimental models. However, the precise role of each HAS isoenzyme in breast cancer progression remains unknown; therefore, the identification of HAS1, -2, and -3 specific regulators should be a priority to avoid a general alteration of HA homeostasis.

The finding that HA accumulates in aggressive breast cancers would require a thorough investigation regarding the identification of which cells (i.e., neoplastic or cancer-associated) synthesize such HA. For example, it is well known that in pancreatic tumors, cancer cells use HA as an energy source, exploiting the production of HA by stromal cells [193]. Therefore, a deep understanding of the crosstalk among different cell types in the tumor microenvironment is of great importance.

Future approaches to breast cancer could involve engineered immune cells (currently used for hematological malignancies) or molecules capable of starving tumor cells, such as those inhibiting amino acid metabolism (i.e., glutamine antagonists). HA coating around cancer cells can mask critical epitopes for immune cell recognition, and the starving approach could reduce HA synthesis. Therefore, new combined approaches involving HA inhibition should be considered to maximize the efficacy of future therapies.

## 7. Conclusions

Breast cancer is a highly heterogeneous malignancy with variable morphological, biological, and clinical features, and an efficient response to treatment is challenging. The discovery of the crucial role of hormone receptors in breast cancer cells has revolutionized therapies, allowing targeted approaches. However, it is evident that the tumor microenvironment plays a fundamental role in directing cancer-associated cells, infiltrating immune cells, vascular cells, and fibroblasts toward protumorigenic or antitumorigenic effects. HA is a ubiquitous component of the ECM in cancer and healthy tissues. Although mainly associated with aggressive ER-negative tumors, HA and HA metabolic enzymes play a critical role in breast cancer cell proliferation, metabolism, chemoresistance, and metastasis, and inhibition of HA synthesis with 4-MU could represent a new pharmacological target for the treatment of these cancers within the next few years. The study of new and specific regulators of HA metabolism, such as HAS2-AS1, could represent new strategies for treating this type of cancer in the future. Interestingly, the HA receptor CD44 is highly expressed in the subpopulation of breast cancer cells with stem cell potential, and HA-coated particles could be used as a vehicle to reach such an aggressive population of cells.

## Figures and Tables

**Table 1:** Molecular profiles and expression of HA-related gens ER^+^ vs. ER^−^ breast cancer cell lines.

	MDA-MB-231	Hs 578T	SUM149	BT-549	MCF-7	T-47D	BT-474
TUMOR ^1^	AC	IDC	InfDC	IDC	AC	IDC	IDC
ER ^2^	-	-	-	-	+	+	+
PR ^2^	-	-	-	-	+	+	+
HER2 ^2^	-	-	-	-	-	-	+
HAS1 ^2^	±	±	±	±	+	±	±
HAS2 ^2^	+	+++	+	+	±	±	±
HAS3 ^2^	+	+	+	++	±	±	±
HAS2-AS1 ^3^	++	+++	++	+++	±	-	-
UGDH ^2^	++	++++	+	++	++++	+	++
HYAL1 ^2^	±	+	+	+	±	+	±
HYAL2 ^2^	+	++	+	++	+	+	+
RHAMM ^2^	+	+	+++	+++	+	++	+
CD44 ^2^	++++	++++	++++	++++	+	+	+
CEMIP ^2^	++	++++	+	+	+	+	+
TMEM2 ^2^	+	++	+	+	+	+	+

^1^ Information from ATCC, ^2^ the human protein atlas and ^3^ [90]. AC, adenocarcinoma; IDC, invasive ductal carcinoma; InfDC, infiltrating ductal carcinoma. +/- data refer to normalized transcript per million (nTPM). -, 0 nTPM; ±, 0–1 nTPM; +, 1.1–50 nTPM; ++, 50.1–100 nTPM; +++, 100.1–200 nTPM; ++++, >200 nTPM.

## Data Availability

Figures presented in this review were created with BioRender.com, accessed on 29 June 2023, with agreement numbers TC25MV1E8N and WX25MUW9GU to A.P. (Arianna Parnigoni).
